# Low-temperature deposition manufacturing technology: a novel 3D printing method for bone scaffolds

**DOI:** 10.3389/fbioe.2023.1222102

**Published:** 2023-08-09

**Authors:** Tianze Sun, Jinzuo Wang, Huagui Huang, Xin Liu, Jing Zhang, Wentao Zhang, Honghua Wang, Zhonghai Li

**Affiliations:** ^1^ Department of Orthopedics, First Affiliated Hospital of Dalian Medical University, Dalian, China; ^2^ Key Laboratory of Molecular Mechanism for Repair and Remodeling of Orthopedic Diseases, Dalian, Liaoning, China; ^3^ Division of Energy Materials (DNL22), Dalian Institute of Chemical Physics, Chinese Academy of Sciences, Dalian, China

**Keywords:** three-dimensional printing, low-temperature deposition manufacturing, rapid prototyping manufacturing, bone tissue engineering, scaffold

## Abstract

The application of three-dimensional printing technology in the medical field has great potential for bone defect repair, especially personalized and biological repair. As a green manufacturing process that does not involve liquefication through heating, low-temperature deposition manufacturing (LDM) is a promising type of rapid prototyping manufacturing and has been widely used to fabricate scaffolds in bone tissue engineering. The scaffolds fabricated by LDM have a multi-scale controllable pore structure and interconnected micropores, which are beneficial for the repair of bone defects. At the same time, different types of cells or bioactive factor can be integrated into three-dimensional structural scaffolds through LDM. Herein, we introduced LDM technology and summarize its applications in bone tissue engineering. We divide the scaffolds into four categories according to the skeleton materials and discuss the performance and limitations of the scaffolds. The ideas presented in this review have prospects in the development and application of LDM scaffolds.

## Graphical Abstract

**Figure:**
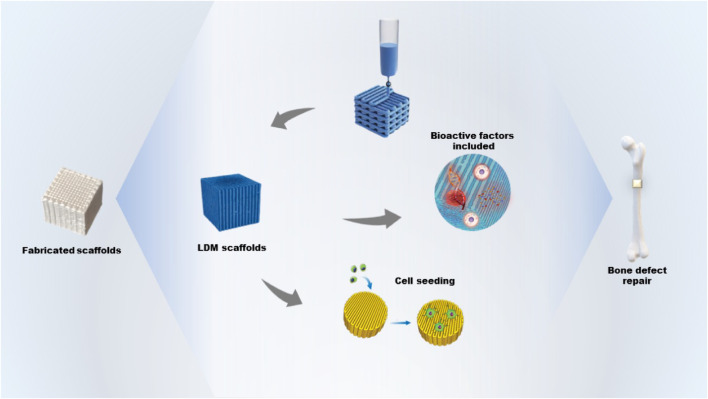
The fabrication of bone tissue engineering scaffolds including bioactive factors by low-temperature deposition manufacturing and its application for bone defect repair.

## 1 Introduction

Bone defects, mostly those concerning a young and athletic population, are increasingly receiving attention (Sfeir et al., 2022). In general, bone tissue has a natural capacity to regenerate, which helps the repair of minor injuries. However, large bone defects due to pathological fractures or high-energy injuries present a clinical challenge requiring bone grafting to overcome. Bone tissue engineering, combined scaffolds, seed cells, and cytokines play important roles in bone repair. The ideal bone tissue engineering scaffold should have a suitable surface for cell attachment, a porous structure for vascularization, and a suitable mechanical support (Roseti et al., 2017). Various technologies for fabricating scaffolds with controlled structure and pore size, including rapid prototyping manufacturing (RPM), gas foaming, and electrospinning, have been reported. Representative fabrication methods and materials of bone tissue engineering are given in Table 1. As an innovative material processing approach, RPM scaffolds have been used in preclinical studies (Dadhich et al., 2016; Yang et al., 2017; Dadhich et al., 2021). The materials of RPM can be liquid or solid, and the printing processes using wet materials include inkjet printing, stereolithography, and direct ink writing. With the help of computer-aided design (CAD) tools, RPM offers the possibility of fabricating complex structures through the layer-by layer deposition of inks of various materials (Jones, 2012; Do et al., 2015; Wubneh et al., 2018). Undoubtedly, RPM is important to the future of bone regeneration for its ability to control the geometry and internal porous structures of scaffolds. In 1999, Leu et al. reported rapid freezing prototyping in milestone work (Zhang et al., 1999; Leu et al., 2000). During the fabricating process, water is deposited from a nozzle in a cryogenic atmosphere and rapidly frozen layer by layer. As an extension, RPM is often called low-temperature deposition manufacturing (LDM) when the injection devices extrude materials used for tissue engineering.

**TABLE 1:** Representative fabrication methods and materials in bone tissue engineering.

Method	Properties		Representative materials
	Advantages	Disadvantages	
FDM	Commonly used and low cost; Broad resource of material and high mechanical strength	Low accuracy and details; Biomaterial restriction due to need for high temperature	TCP, PCL, Al_2_O_3_, TCP/PP, TCP/PCL
Binder jetting	Accuracy and flexibility; Low cost of materials and binders; Low to high temperature allowed	Slow printing speed; Risk of toxicity	HA, α-TCP, β-TCP, PLA, PEG, PLGA, Gelatin, Chitosan
SLS	Suit for polymer-ceramic composites; Fast printing speed; Strong functional parts	Limited material options; resolution depends on laser; Require depowering	PCL, PEEK, PLLA, PGA, PCL/HA, PHBV/HA
SLA	Relatively fast and high resolution; High accuracy; Complex internal features	Sensitive to long exposure; Only applicable for photopolymers	PPF/DEF, PDLLA/HA, β-TCP
Gas foaming	Relatively faster manufacturing processes; High porosity scaffold	Inability to create fully interconnected pores; Inability in creating intricately shaped scaffolds	PCL, PLA, PLLA, PLGA, PLA/HA
Electrospinning	Capable of producing nanofibers; Commonly used for wound dressing	Difficult to control; Fiber size and density are not ideal for guiding bone cell growth	PCL, PGA, PCL/PGA, PCL/HA
			PCL/HA/collagen
LDM	Good porosity; keeping activity of biological factors; Open to blending to a certain extent	Mechanical properties are usually poor	PCL, PLGA, TCP, HA, Gelatin, PLGA/HA

FDM, fused deposition modeling; SLS, selective laser sintering; SLA, stereolithography; TCP, tricalcium phosphate; PCL, poly(ε-caprolactone); HA, hydroxyapatite.

PLA, polylactic acid; PEG, polyethylene glycol; PLGA, poly (lactic-co-glycolic acid); PEEK, polyetheretherketone; PLLA, poly (L-lactic acid); PGA, polyglycolic acid.

PHBV, poly (3-hydroxybutyrate-co-3-hydroxyvalerate); PPF, poly(propylene fumarate); DEF, diethyl fumarate; PDLLA, poly-DL-lactide.

LDM was firstly reported in 2002 for the fabrication of bone tissue engineering scaffolds, which is based on traditional fabrication technologies such as direct ink writing (Xiong et al., 2002). The material used in LDM is usually a viscous polymer and the extruder is usually of piston or pneumatic type operating at room temperature (Geven and Grijpma, 2019). In addition, scaffolds can be fabricated on a cold platform or in a freezer layer-by-layer with the solvent then removed by freeze drying (Papastavrou et al., 2018; Qin et al., 2021). Compared with conventional technologies, LDM is combined with a phase separation process and the scaffolds have a hierarchically porous structure from microns to nanometers that is beneficial to cell adhesion and tissue growth (Liu et al., 2017). However, the mechanical properties of the LDM scaffolds are usually slightly weaker than those of traditional scaffolds. In addition, LDM is a kind of green manufacturing because it does not require heating during fabricating (Zhang et al., 2008). LDM scaffolds thus maintain the bioactivities of natural biopolymers, such as gelatin, chitosan and sodium alginate and are often used for bioprinting or printing tissue (Wang et al., 2012; Zafeiris et al., 2021; Zhao et al., 2022). As LDM is a molding method based on material extrusion, it has requirements for the viscosity of the extruded inks. The ability to control the pore size by selecting the ratio of solvent or precursor solution within the specific viscosity range of each material is an obvious advantage of LDM. Inorganic particles such as nano-hydroxyapatite and tricalcium phosphate (TCP) have been widely used for the backbone structure of scaffolds (Wang et al., 2014; He et al., 2016; Cheng et al., 2021). Moreover, synthetic biopolymers, such as poly- (lactic-co-glycolic acid) (PLGA), poly-(L-lactic acid) (PLLA), and polyurethane (PU), have been commonly used in the fabrication of porous bone tissue engineering scaffolds (Wang et al., 2013; Gentile et al., 2014; Zhang et al., 2022; Zhao et al., 2022). Ideal scaffolds can be fabricated by adjusting the properties of the material, the proportions of composite materials or the printing parameters. LDM has been adopted for bone tissue engineering scaffolds for two decades. Herein, we discuss LDM technology and review its applications in the field of bone tissue engineering.

## 2 Technical process of LDM

### 2.1 Material preparation and optimization

Typically, the LDM material is fully dissolved in an organic solvent, such as 1,4-dioxane, and the ink is mixed evenly adopting an emulsion stabilizer and ultrasonic technology. Then, the ideal material properties are obtained by adjusting the appropriate mass ratio of the different materials and the mixture is stirred well to form a uniform liquid paste (Lai et al., 2018). The concentration of a material in the solvent affects microstructure of scaffolds (Liu et al., 2007). When the polymeric materials are dissolved at higher concentrations in the solvent, the scaffolds have a smaller pore size and thicker walls of micropores. In contrast, scaffolds with lower concentrations of polymeric materials have larger pores and thinner walls of micropores. Hu et al. (2015) fabricated a pure PLGA scaffold, a PLGA/pearl scaffold with a weight ratio of 5:2, and a PLGA/TCP scaffold with a weight ratio of 5:2 and found that the proportions of the composite materials affected the structure of scaffolds in terms of the continuity of pores. Guo et al. (2017) used PLGA with various lactic acid: glycolic acid molecular weight ratios to demonstrate the dependence of the extrusion process on the polymer composition. They built a statistical model to reveal the correlation and predominant factors that determine printing precision.

Synthetic materials have better mechanical properties whereas natural materials have better biocompatibility in scaffolds (Rahmanian-Schwarz et al., 2014). Unfortunately, no solvent dissolves synthetic and natural materials together in LDM. Gelatin or GelMA hydrogel has a thermo-reversible sol-gel property, having a liquid state at 37°C and a gel state at a temperature lower than 20°C (Luo et al., 2020). They can help combine drugs, essential elements, and other bioactive factors with the scaffold material. GelMA also has a photocrosslinking ability that stabilizes the structures after printing. The bioink then enters a sol-gel state quickly and is transferred to a syringe for subsequent three-dimensional (3D) hybrid printing at low temperature.

### 2.2 Printing process and improvements

**FIGURE 1:**
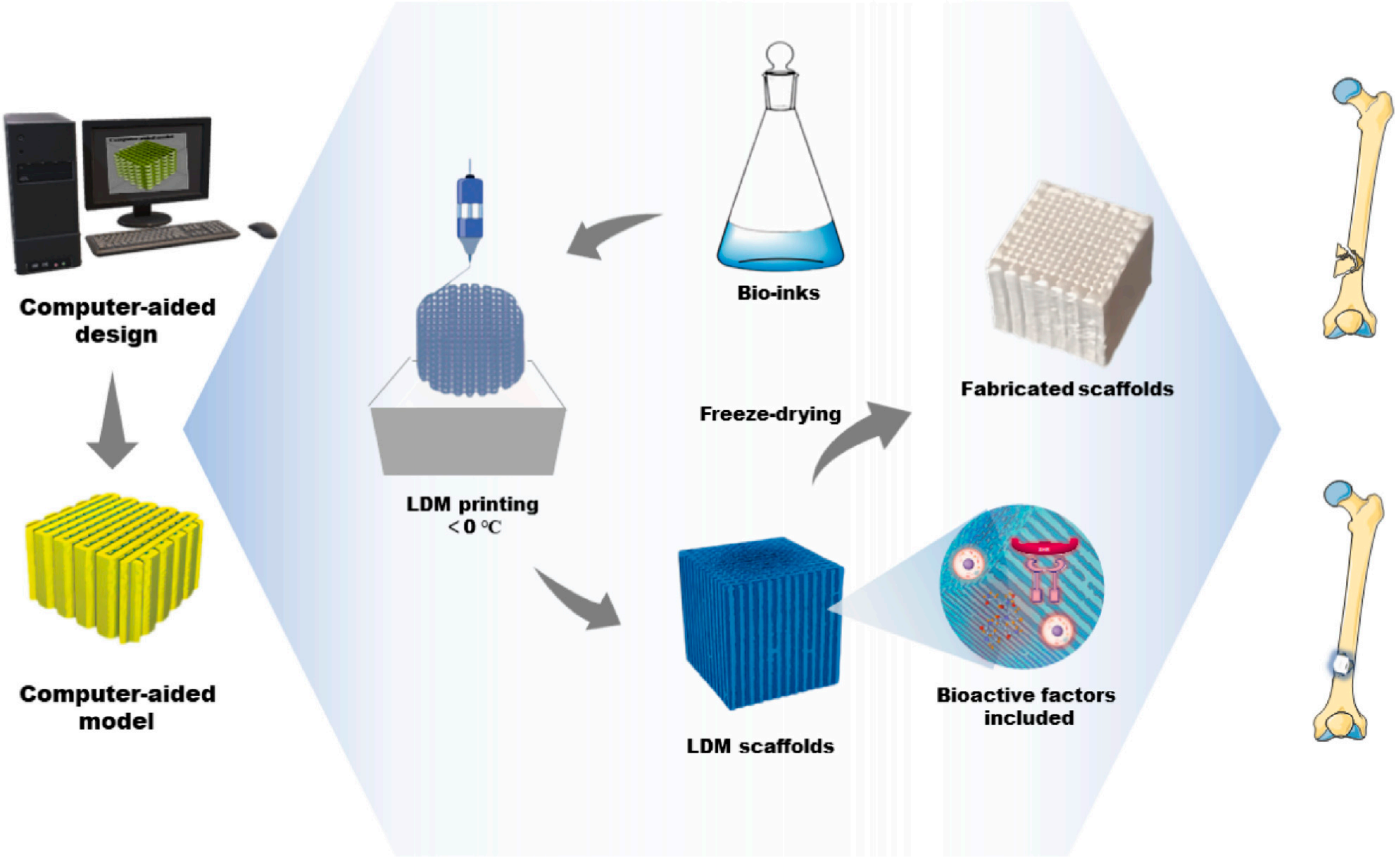
The fabrication process of bone tissue engineering scaffolds by LDM and its application for bone defect repair. LDM, low-temperature deposition manufacturing.

Usually, the structure and pore size of scaffolds determine the cell growth and regeneration of bone tissue. Material properties can be optimized by adjusting the parameters of the LDM system to print more ideal bone tissue engineering scaffolds. The parameters of the LDM system include the proportion of the composite material, the design of the model, the concentration of the material and the working parameters of the devices (Liu et al., 2007; Hu et al., 2015; Bružauskaitė et al., 2016; Wang et al., 2017b; Guo et al., 2017). According to the selection and combination of ink materials, the printer is designed to have a single nozzle or multiple nozzles, and is used with the corresponding syringe (Liu et al., 2009a; Liu et al., 2009b). In the process of LDM, the shape and architecture of scaffolds are controlled through freeform fabrication and the features of microstructure, such as porosity and surface roughness, are realized manufactured through freezing drying (Koski et al., 2018). The dispensing system deposits printing inks in a low-temperature environment, which is a continuous extrusion process in contrast with inkjet processes. The ink-containing syringe extrudes the ink through a micro-nozzle. Compared with other traditional techniques, LDM based on extrusion has an appropriate deposition and printing speed during printing, which facilitates rapid scalability (Johnson and Jia, 2016; Lee et al., 2020). Figure 1 depicts the LDM process of bone tissue engineering scaffolds and the application for bone defect repair. The advantage of this method is the variety of options of printing inks. In bone tissue engineering, various materials containing biologics have been successfully applied for the fabrication of tissue engineering scaffolds (Mehesz et al., 2011; Kronemberger et al., 2022). During printing, the deposited ink is cured to strengthen each layer. As the paste freezes, its particles are expelled from the solidification (freezing) front (Papastavrou et al., 2018). The frozen part is freeze-dried to sublimate the freezing medium and leave a highly porous scaffold.

The shape and architecture of scaffolds depend on the models designed using computer software (Bružauskaitė et al., 2016). CAD tools offer the possibility to fabricate complex structures and custom scaffolds. In addition, the working parameters of LDM printers need to be adjusted for the fabrication of ideal scaffolds. As an example, the forming platform usually remains below a temperature of 0°C and the temperature of the nozzles must be much higher than that of the platform. Only in this way can the extruded lines be integrated with the previous layer. The extrusion speed and nozzle diameter determine the structure and size of scaffolds (Wang et al., 2017b). In the process of printing, it is necessary to control the appropriate temperature of the platform and nozzle according to the material properties and surrounding environment. At the same time, the temperature, pressure, speed, and other parameters need to be constantly adjusted to ensure the smooth printing of the scaffolds.

## 3 LDM materials for bone tissue engineering scaffolds

### 3.1 LDM scaffolds of bio-ceramic materials

Hydroxyapatite (HA) and other related Ca/P-based bio-ceramics have been used in the manufacturing of bone scaffolds for their varying osteoconductive and osteoinductive properties. They not only exist naturally in bone tissue but also have high mechanical strength and biodegradability. Moreover, the biodegradation rates can be optimized by adjusting the molar ratio of Ca and P in the optimized compound. In addition, the inorganic materials can be categorized into silicate-based glasses, borate-based glasses and phosphate-based glasses according to the components of the bioactive glass (Simorgh et al., 2022). The majority of bio-ceramic materials are subjected to a sintering procedure and high temperature treatment to prepare the scaffolds and achieve sufficient mechanical qualities (Baino and Fiume, 2020). As an example, hydrogel, containing active elements is often mixed physically with the scaffold to provide the ceramic material bioactivity (Simorgh et al., 2022). In addition, several studies have used bio-ceramic materials to formulate inks for the preparation of bone tissue engineering scaffolds through LDM. Table 2 gives bone tissue engineering scaffolds based on bio-ceramic materials. We divide the scaffolds with bio-ceramic skeletons into bio-ceramic scaffolds, bio-ceramic scaffolds with natural polymers, and bio-ceramic scaffolds with bioactive factors.

**TABLE 2:** LDM of bio-ceramic skeleton scaffolds for bone tissue engineering.

Year	Team	Materials	Properties of scaffolds
2004	Almirall et al	α-TCP/CDHA	The scaffolds have larger porosity and biological surface while the mechanical strength is low
2010	Klammer et al	Mg3(PO4)2/DAHP	STL data helped design the scaffolds and DAHP helped to improve the mechanical strength
2012	Moseke et al	β-TCP/MCPM	The hardened granules were microporous and consisted of crystals of 0.5–7 μm size
2013	Castiho et al	TCP/phosphoric acid	The scaffolds were optimized adopting computer and considered permeability during fabrication
2014	Inzana et al	Calcium Phosphate/Collagen	The mechanics were strengthened adopting increasing the concentration of binder solution
2016	Lin et al	Collagen/HA	The CHA scaffolds had excellent 3D structure and promoted cell proliferation and osteogenic outcome
2016	Raina et al	HA/SF/Chitosan/BMP-2/ZA	Bio-composite cryogels as carrier scaffolds for bone active agents augmenting bone regeneration
2018	Huh et al	Gelatin/TCP/SF	The scaffolds had enhanced mechanical properties and high cellar activity
2018	Ahlfeld et al	CPC/alginate-methylcellulose	The bio-ink was cell-laden and fabricated constructs with spatially defined cell distribution
2018	Lee et al	HA/collagen/PRP	The scaffolds had growth factors and a polyphenol tannic acid was used to control the release of PRP.
2020	Chen et al	HA/BMP-2/VEGF	The scaffolds showed well porous structure and better osteogenic but the compressive strength was low
2020	Bolaños et al	α/β-TCP/phosphoric acid	The scaffolds had excellent ability to promote bone formation and showed well mechanical properties
2021	Liu et al	HA/SF/Gelatin	The compressive modulus of scaffolds was enhanced and had better cell proliferation and osteogenesis
2021	Zafeiris et al	HA/Chitosan	The scaffolds were Chemical crosslinked to improve mechanical strength close to cancellous bone

TCP, tricalcium phosphate; CDHA, calcium deficient hydroxyapatite; DAHP, diammonium hydrogen phosphate; MCPM, mono calcium phosphate monohydrate.

BCP, biphasic calcium phosphate; HA, hydroxyapatite; PRP, plate-rich plasma; SF, silk-fibroin; CPC, calcium phosphate; BMP-2, bone morphogenetic protein-2.

ZA, zoledronic acid; VEGF, vascular endothelial growth factor.

#### 3.1.1 Bio-ceramic scaffolds

**FIGURE 2:**
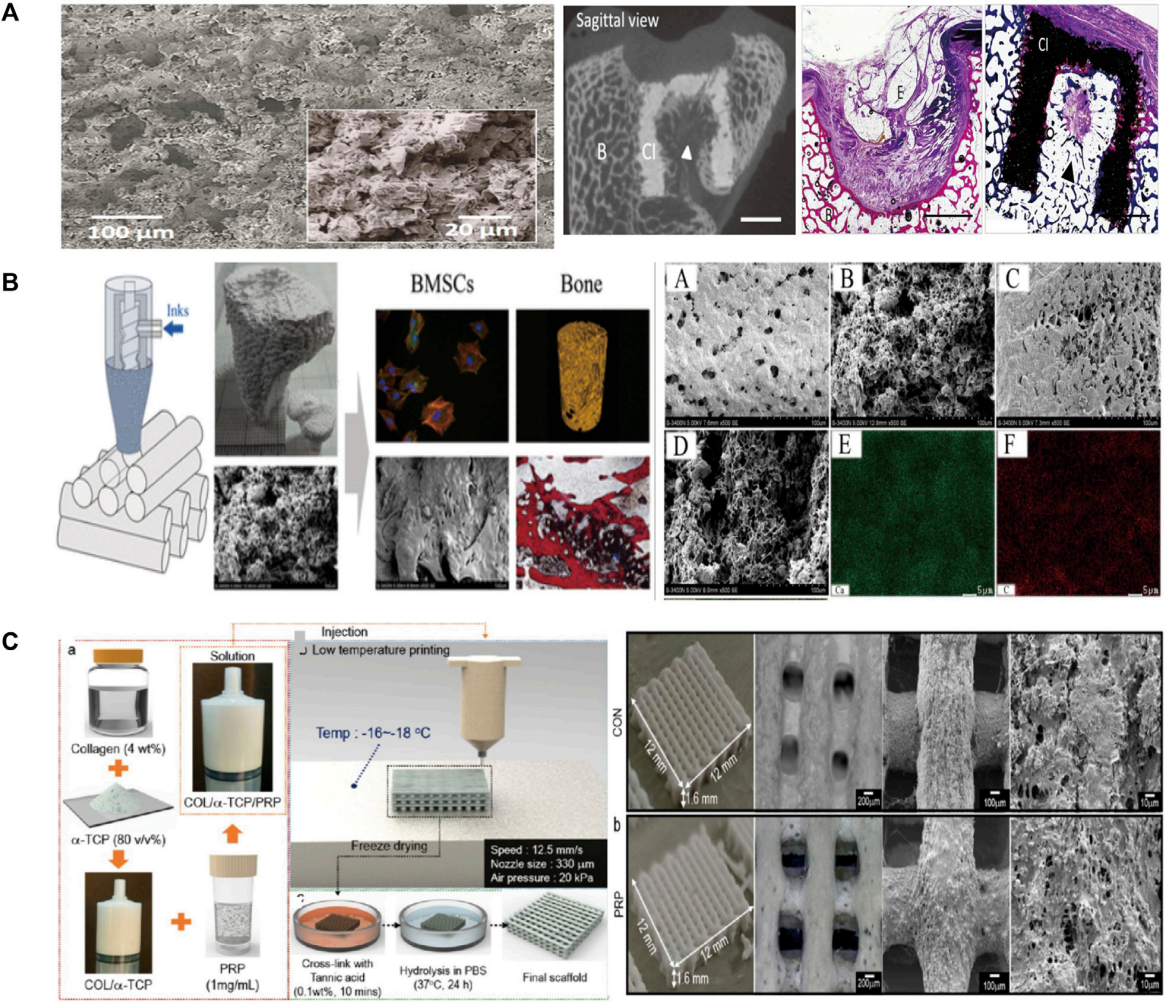
**(A)** Microscopic morphology and *in vivo* evaluation of the scaffolds. **(B)** The robocasting fabrication process and SEM images of the surface morphology. Micro-CT 3D reconstruction images and histological analysis of new bone formation around and within the scaffolds. **(C)** Schematic of the preparation of scaffolds, as well as the macro/micro morphology shown by optical and SEM images. SEM, Scanning electron microscope.

Klammert et al. (2010) used binder solution and powder (Mg_3_(PO_4_)_2_) to print scaffolds at room temperature and dried the scaffolds for 24 h. The structures were designed on the basis of stereolithography data and modified with 20% DAHP powder to improve the conversion rate and mechanical reinforcement (Figure 2A). However, they did not conduct *in vivo* investigations to explore other biological features. Castilho et al. used TCP powder and phosphoric acid solution as a binder to print dense cylindrical and porous scaffolds at room temperature (Castilho et al., 2013). They optimized the computational topology design to better fabricate the scaffolds through LDM and also considered the mechanical properties and permeability. Bolaños et al. printed tailor-made ceramic scaffolds with α/β-TCP and phosphoric acid binder at low temperature (Bolaños et al., 2020). An *in vivo* experiment using a large animal model showed that the material was degraded and replaced by bone, and the scaffolds had an excellent ability to promote bone formation. At the same time, *in vitro* experiments showed that porous implants had good mechanical properties.

#### 3.1.2 Bio-ceramic scaffolds with natural polymers

The brittleness of scaffolds is a main shortcoming of ceramic material. Bio-additives, such as collagen and silk fibroin (SF), are naturally degradable and can be used to optimize the ceramic scaffolds. HA and collagen are often added to the precursor solution to improve the mechanical and clinical properties of the scaffolds (Lin et al., 2016; Salgado et al., 2016). The scaffolds prepared at 4°C by Lin et al. (2016). had an excellent 3D structure and enhanced the osteogenic outcome in rabbit femoral condyle defect models (Figure 2B) (Lin et al., 2016). Huh et al. (2018) prepared bio-ink by mixing gelatin, TCP, and SF loaded the solution into a barrel with the temperature controlled from 25°C to 45°C and printed on the low temperature stage of 10°C (Huh et al., 2018). The scaffold had enhanced mechanical strength and high cellar activity. Liu et al. (2021b) used HA, SF, and gelatin to prepare bio-inks and fabricated scaffolds adopting crosslinking and freeze-drying technologies. The scaffolds were cross-linked under absolute alcohol at room temperature for 24 h. They found that the scaffolds enhanced the compressive modulus in two groups and were conducive to cell proliferation and osteogenesis. Zafeiris et al. (2021) fabricated hydrogels by HA and chitosan and then adopting chemical crosslinking in a natural crosslinking agent to improve mechanical properties. The syringe was prepared with a nozzle tip diameter of 0.41 mm and a printing speed of 0.8–1.5 mm/s to improve the printing accuracy. The 3D printing process was applied at a low temperature of 25°C and the scaffolds were freeze dried to obtain a porous structure by removing the solvent. They obtained mechanical properties close to those of cancellous bone and good cell compatibility. Inzana et al. (2014) increased the phosphoric-acid-based binder solution concentration and dissolved collagen into the binder to maximize mechanical strength and cytocompatibility. This method enhanced the mechanical strength of materials without reducing biocompatibility. They also implanted calcium-phosphate-based scaffolds into murine femoral defect to assess the bone healing performance and osteoconductive.

#### 3.1.3 Bio-ceramic scaffolds with bioactive factors

Another disadvantage of ceramic material is the low retention level of growth factors and drugs. Therefore, bioactive substances such as live cells and growth factors are integrated in bioinks to increase the biological activity and osteogenic ability of bone repair materials. It is not a difficult task to impregnate biological factors into the mixed bioink and the low temperature avoid the risk of thermal degradation of drugs or growth factors. To overcome the shortcomings, Lee et al. designed a new composite with stable structure using CDHA, collagen, and plate-rich plasma (PRP) to enable better growth and differentiation of cells (Lee and Kim, 2018a). They used a nozzle size of 330 μm to print scaffolds at a low temperature of −16°C. Figure 2C (Lee and Kim, 2018a) is a schematic of the preparation of scaffolds and presents the macro/micro-morphology in optical and scanning electron microscopy images. The PRP had several growth factors, and a polyphenol tannic acid was used to control the release of PRP. The result *in vitro* showed that PRP composited scaffolds have better bone mineralization and cell proliferation, but the efficacy for bone regeneration *in vivo* remains controversial. Ahlfeld et al. (2018) combined the 3D plotting of calcium phosphate and an alginate-methylcellulose cell-laden blend as model bioink . They printed scaffolds were printed at room temperature using a needle with an inner diameter of 410 µm and a layer thickness of 250 µm. They proposed an osteochondral tissue graft model and evaluated the mechanical properties of the scaffolds. They found that bone morphogenetic protein-2 (BMP-2) promotes osteogenesis and vascular endothelial growth factor promotes angiogenesis. Chen et al. (2020) prepared composite scaffolds loaded with BMP-2 and vascular endothelial growth factor through 3D printing layer by layer at a temperature below 40°C. The nozzle of the printer had a diameter of 0.42 mm and the distance between strands was set at 600 μm to ensure good printing accuracy. They found that the scaffolds had good porosity and osteogenic and angiogenic properties through microcomputed tomography and immunochemical staining. However, the interaction of chitosan with gelatin achieved a compressive strength of only 6 MPa, which is below many clinical requirements. Similarly, Raina et al. formulated a precursor solution by mixing rhBMP-2 and bisphosphonates such as zoledronic acid, which can be used to control the resorption process (Raina et al., 2016). The scaffold loaded with both rhBMP-2 and zoledronic acid showed high bone formation.

### 3.2 LDM scaffolds of synthetic polymeric materials

Compared with ceramics, polymeric materials have more versatile physicochemical properties that suit clinical applications. Synthetic polymers usually have good processability and mechanical properties and are thus used widely in preparing materials for bone tissue engineering technology (Lutolf and Hubbell, 2005; Rezwan et al., 2006; Amiryaghoubi et al., 2020). Table 3 gives bone tissue engineering scaffolds based on polymeric materials. According to the compositions and functions, we divided the scaffolds with synthetic polymeric skeletons into three categories, namely, synthetic polymeric scaffolds with bioactive factors, synthetic polymeric scaffolds with hydrogels and synthetic polymeric scaffolds with bioactive factors/hydrogels or modified polymers.

**TABLE 3:** LDM of polymer skeleton scaffolds for bone tissue engineering.

Year	Team	Materials	Properties of scaffolds
2006	Fan et al	PLGA-gelatin/chondroitin/hyaluronate	The scaffolds showed better proliferation and differentiation of MSCs for bone and cartilage repair
2010	Xu et al	PLGA/Pearl	The scaffolds had high porosity, proper pore size and mechanical property
2016	Kim et al	Alginate/Collagen/PCL	The tensile modulus, cell proliferation and deposition of calcium were significantly increased
2018	Kim et al	Gelatin/PVA	The scaffolds showed better physical and biological properties through various weight fractions of PVA and gelatin
2018	Wang et al	PU/SPIO NPs/PEO	The scaffolds were biodegradable and showed better shape memory properties and cell viability
2018	Guo et al	PLGA/BMP-2/TGF-β	The bioactive factors were dissolved in bio-ink adopting dimethyl sulfoxide and showed more stretchability
2018	Lee et al	Collagen/PF-127	The scaffolds had fully interconnected macropores and had properties of thermoreversible polymer
2019	Zhang et al	PLGA/DACECM	The scaffold had rough surface and a three-dimensional structure with interconnected pores
2021	Chen et al	WPU/ECM	WPU improved the mechanical properties and ECM improved the bioactivity and porosity of the scaffolds
2021	Geng et al	PGSLP/Gelatin/DFO	The microporous scaffolds promoted the process of endothelial cell migration and osteoblast differentiation
2021	Long et al	PLGA/Mg	The scaffolds had biomimetic hierarchical porous structures and released Mg ions to promote bone regeneration
2022	Gao et al	PAEK-COOH	The scaffolds had hierarchically porous and the mineralization was induced by electrostatic of carboxyl groups
2022	Zhang et al	SMPU/Mg	The scaffolds had porous structure, improved mechanical properties and stable photothermal effects
2022	Zhao et al	PLLA/Alginate/ibuprofen/Sr	The scaffolds had enhanced mechanical stability, well osteogenic activity and anti-inflammatory activity

PLGA, poly (lactic-co-glycolic acid); MSCs, mesenchymal stem cells; PCL, poly(ε-caprolactone); PVA, poly (vinyl alcohol); PU, polyurethane; SPIO NPs, superparamagnetic iron oxide nanoparticles; PEO, polyethylene oxide; BMP-2, bone morphogenetic protein-2; TGF-β, transforming growth factor-β; PF-127, Pluronic F-127; DACECM, decellularized acellular cartilage extracellular matrix; WPU, waterborne polyurethane; ECM, extracellular matrix; PGSLP, poly (glycerol-co-sebacic acid-co-L-lactic acid-co-polyethylene glycol); DFO, deferoxamine; Mg, magnesium; PAEK-COOH, polyaryletherketone with carboxyl groups; SMPU, shape memory polyurethane; PLLA, poly (L-lactic acid).

#### 3.2.1 Synthetic polymeric scaffolds with bioactive factors

**FIGURE 3:**
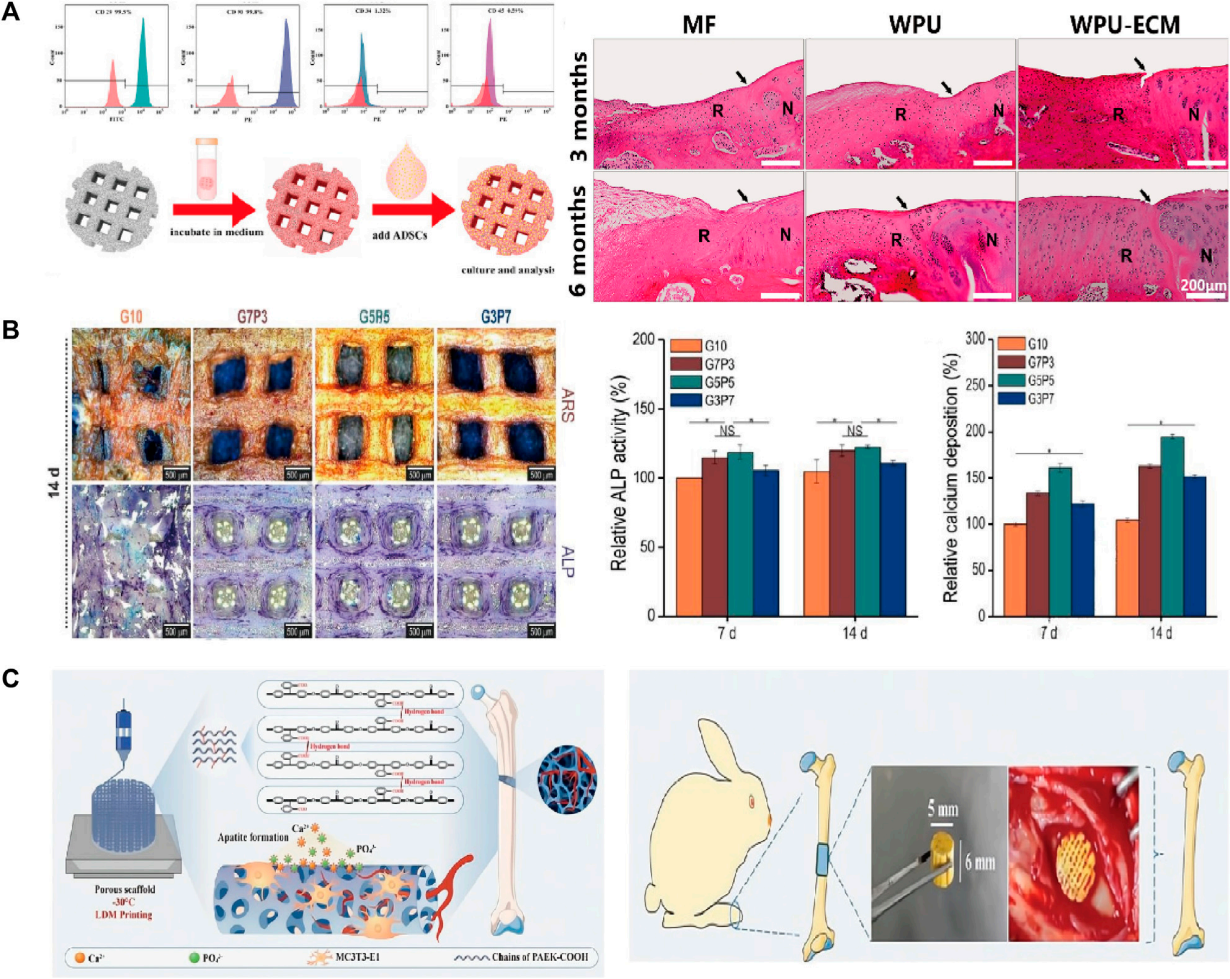
**(A)** Flow cytometric analysis and the schematic diagram of ADSCs seeding. The H&E staining images of the repaired cartilage at 3 and 6 months after cartilage defect creation and scaffold implantation. **(B)** The optical images of ARS and ALP staining of the scaffolds. The quantitative results of ALP activity and calcium deposition. **(C)** The hierarchically porous scaffold of PAEK-COOH favoring cellular adhesion and HA mineralization. The scaffold implanted in rabbit femur defects model induced bone formation. ADSCs, Adipose-derived stem cells; ARS, alizarin red S; ALP, alkaline phosphatase; PAEK-COOH, polyaryletherketone with carboxyl groups.

It is often necessary to composite bioactive factors or modify the materials to increase biocompatibility or osteogenic capacity. PLGA is an excellent biomaterial for bone scaffolds with biodegradability and biocompatibility and has good matrix for pearl powder, which can provide bioactivity for PLGA (Zhang et al., 2009). Xu et al. (2010) used LDM technology to combine the advantages of pearl and PLGA and fabricated scaffolds on a platform at a temperature of −40°C. The scaffolds had a porous structure and good biocompatibility, and gene expression and alkaline phosphatase (ALP) activity tests showed osteo-inductive bioactivity more extensive than that of TCP/PLGA scaffolds. Wang et al. (2018) synthesized shape memory PU to fabricate scaffolds adopting LDM technology. The bioink included superparamagnetic iron oxide nanoparticles to promote osteogenic induction and polyethylene oxide or gelatin to improve printing. In their study, the platform was set at −30°C for PU/polyethylene oxide ink and 5°C for PU/gelatin ink. Dimethyl sulfoxide (DMSO) is a solvent that dissolves both polar and nonpolar compounds (Galvao et al., 2014). Guo et al. (2018) incorporated growth factors such as BMP-2 and TGF-β (transforming growth factor-β) into PLGA scaffold using DMSO. They obtained stretchability greater than that of pure PLGA scaffolds. However, DMSO is cytotoxic and needs to be removed completely through evaporation during the preparation. Zhang et al. (2019) adopted LDM technology to fabricate a PLGA scaffold and compounded with decellularized acellular cartilage extracellular matrix through physical–chemical cross-linking. The scaffolds were printed at −20°C and had good biological and mechanical characteristics, but were deficient in the function of recruiting endogenous stem cells. Chen et al. printed scaffolds with acellular cartilage extracellular matrix and waterborne polyurethane (PU) on a platform at temperatures ranging from −25 to −30°C (Chen et al., 2021). Figure 3A (Chen et al., 2021) presents a schematic of adipose-derived stem cell seeding and hematoxylin and eosin staining images of repaired cartilage at 3 and 6 months after operation. The figure shows that the porosity, hydrophilicity, and bioactive components of the scaffolds are improved by adding the extracellular matrix. In view of the photothermal effect and biological activity of magnesium, researchers composited PLGA and shape memory polyurethane (PU) with Mg respectively (Long et al., 2021; Zhang et al., 2022). They constructed the scaffolds adopting LDM technology and verified the good osteogenic performance of the scaffolds. As the thermal-responsive matrix, shape memory PU significantly improved the mechanical properties of the scaffolds and made closer contact with bone tissue.

#### 3.2.2 Synthetic polymeric scaffolds with hydrogels

Hydrogels have strong hydrophilicity, that provides an environment suitable for cell proliferation and differentiation. Therefore, the biological activity and mechanical properties of the scaffold can be improved by compositing hydrogel and polymer. For example, a novel composited PLGA-gelatin/chondroitin/hyaluronate scaffold was fabricated to keep the differentiation of mesenchymal stem cells (MSCs) and improved the regeneration of cartilage (Fan et al., 2006). Kim et al. (2016) proposed a 3D printing method with a low temperature working plate to fabricate scaffolds. They directly printed alginate and collagen layer by layer on a low-temperature (−20°C) cooling plate using a 250-µm printing nozzle and poly (ε-caprolactone) (PCL) as the coating agent. They found that the tensile modulus and osteogenic capacity of the scaffolds were better than those of the pure-hydrogel scaffolds. However, the strong cooling effect of the low-temperature working plate limited the height of the manufactured porous scaffolds, which introduced defects in this LDM process. Similarly, another study reported that adding electrospun PCL fibers into a gelatin hydrogel solution increased the Young’s modulus of the resulting construct (Kai et al., 2012). Another team fabricated scaffolds with gelatin and poly (vinyl alcohol) adopting LDM process (Kim et al., 2018). They explored the best mixture ratio to get the optimal mechanical and biological properties for a working stage temperature ranging from −5 to −40°C. The quantitative results of ALP activity and calcium deposition and optical images of ALP staining of the scaffolds are shown in Figure 3B (Kim et al., 2018). Lee and Kim (2018b) developed scaffolds having a multilayered nanofibrous structure and composited with collagen and pluronic F-127. Geng et al. (2021) fabricated a biodegradable poly (glycerol-co-sebacic acid-co-L-lactic acid-co-polyethylene glycol) scaffold and filled it with gelatin nanofibers. The scaffold promoted bone repair by locally releasing deferoxamine, which is essential for angiogenesis and osteogenesis.

#### 3.2.3 Synthetic polymeric scaffolds with bioactive factors/hydrogels or modified polymers

Zhao et al. dispersed PLLA containing ibuprofen into sodium alginate aqueous solution to prepare the bioink (Zhao et al., 2022). The scaffolds were fabricated at a temperature of −20°C using a 0.41 mm printing nozzle. The plotting pressure was 130 kPa and the extrusion speed was fixed at 3 mm/s. The scaffolds had enhanced mechanical stability, excellent osteogenic activity, and anti-inflammatory activity. In contrast with work that composited bioactive factors and hydrogels, Gao et al. (2022) synthesized an amorphous polyaryletherketone with carboxyl groups (PAEK-COOH). They fabricated scaffolds by LDM technology at a temperature of −30°C, where the porosity was hierarchically controlled and the implanted scaffold induced bone formation *in vivo* (Figure 3C) (Gao et al., 2022). Compared with other degradable materials, the scaffolds had high mechanical strength that made up for the poor mechanical properties of the low-temperature solution printing method. The electrostatic interaction of carboxyl groups induced HA mineralization and the porous surface further promoted cell adhesion. This research was a breakthrough in the field of bone regeneration and repair of polymeric materials, and research on combining the active factors of PAEK-COOH is currently underway.

### 3.3 LDM scaffolds of polymer-ceramic materials

High-molecular-weight polymers usually have poor geometric properties, such as a poor pore size and poor porosity and interconnection, which affect the attachment of scaffolds to cells. Generally, the ability of a polymer to support bone conduction is improved by adding ceramic filler to form a polymer–ceramic composite scaffold. Compared with pure polymer, ceramic composites have obvious benefits in terms of cell performance. Table 4 presents bone tissue engineering scaffolds with polymer–ceramic skeletons. We divide the scaffolds into three categories.

**TABLE 4:** LDM of polymer-ceramic skeleton scaffolds for bone tissue engineering.

Year	Team	Materials	Properties of scaffolds
2009	Kai et al	PLGA/TCP/Chitosan	Using computer-assisted design system to optimal parameters based on a double-nozzle technology
2011	Li et al	PLGA/TCP	The scaffolds were identical to defects and the mechanical properties were similar to cancellous bone
2012	Wang et al	PLGA/β-TCP/Collagen	The scaffolds were wrapped with Type I collagen and had greater abilities of proliferation and osteogenic
2013	Chen et al	PLGA/TCP/Icariin	The scaffolds composited with icariin had more mineralized bone and more new blood vessels ingrowth
2013	Chen et al	PLGA/nHA	The results indicated that the viability of cells on the scaffolds were not affected by nHA/PLGA.
2014	Dong et al	PDLLA/HA/Anti-TB drugs	The composited scaffolds were fabricated by solvent evaporation and low temperature drying technology
2015	Yoshida et al	PLGA/β-TCP/FGF-2	The PLGA coating scaffolds had high porosity, good compressive strength and biocompatibility
2015	Wei et al	PLGA/β-TCP/RGD	The scaffolds with the peptide increased the cell proliferation and osteogenic differentiation of the BMSCs
2017	Wang et al	PLLA/CaP/BMP-2	The scaffolds had better mechanical properties and had sustained release of Ca2+ and BMP-2 for osteogenic
2017	Zhang et al	PLGA/TCP/Collagen	The scaffolds were designed to mimic mechanical properties and hydrophilicity of bone and cartilage
2018	Huang et al	Col-I/PLGA/n-HA/Fe2O3	The nanocomposite scaffolds had suitable mechanical properties and compatibility for caricular repairment
2018	Lai et al	PLGA/TCP/Icariin	The scaffolds had a well-designed structure to provide mechanical support and stable icariin release
2018	Song et al	PVA/CaP/PRF	PRF improved biological activities and bioactive factors could sustained release from scaffolds
2019	Lin et al	PLGA/β-TCP/SB	The composite scaffold could enhance osteogenesis and angiogenesis by incorporating with SB.
2019	Lai et al	PLGA/β-TCP/Mg	The scaffold had both osteogenic and angiogenic abilities to enhance the formation of new bone
2020	Diloksumpan et al	PCL/α-TCP/Hydrogel	The hydrogel and CaP were encased in PCL cages to obtain better osteogenic from only one direction
2021	Dou et al	PLGA/nHA/Gelatin	The scaffolds had large front and side hole size to provide space and support for tissue ingrowth
2021	Cheng et al	PLGA/β-TCP/CuB	CuB released from the scaffolds and enhances bone regeneration and neovascularization in the models
2021	Lian et al	PLCL/HA	The scaffolds promoted the adhesion and paracrine of MSCs, thereby improving the osteogenic ability
2021	Zhang et al	PCL/β-TCP/Icariin	The scaffolds had highly porous structure and maintained the bio-efficacy of icariin for tissue regeneration
2022	Lai et al	PLGA/β-TCP/OP/rBMSC	The OP could be released from the scaffolds and enhanced the proliferation and differentiation of BMSC.
2022	Wang et al	PCL/nHA/Gelatin-CaO2	The scaffolds had bionic hierarchical porous structures and could release O2 sustainably for bone repair

PLGA, poly (lactic-co-glycolic acid); TCP, tricalcium phosphate; n-HA, nano-hydroxyapatite; PDLLA, poly-DL-lactide; HA, hydroxyapatite; FGF-2, fibroblast growth factor-2; RGD, arginine-glycine-aspartic acid; PLLA, poly (L-lactic acid); BMP-2, bone morphogenetic protein-2; PVA, poly (vinyl alcohol); SB, Salvianolic acid B; CuB, cucurbitacin B; PLCL, poly (L-lactic acid-ε-caprolactone); OP, osteogenic peptide; rBMSC, rat bone marrow derived mesenchymal stem cell.

#### 3.3.1 Polymer–ceramic scaffolds

**FIGURE 4:**
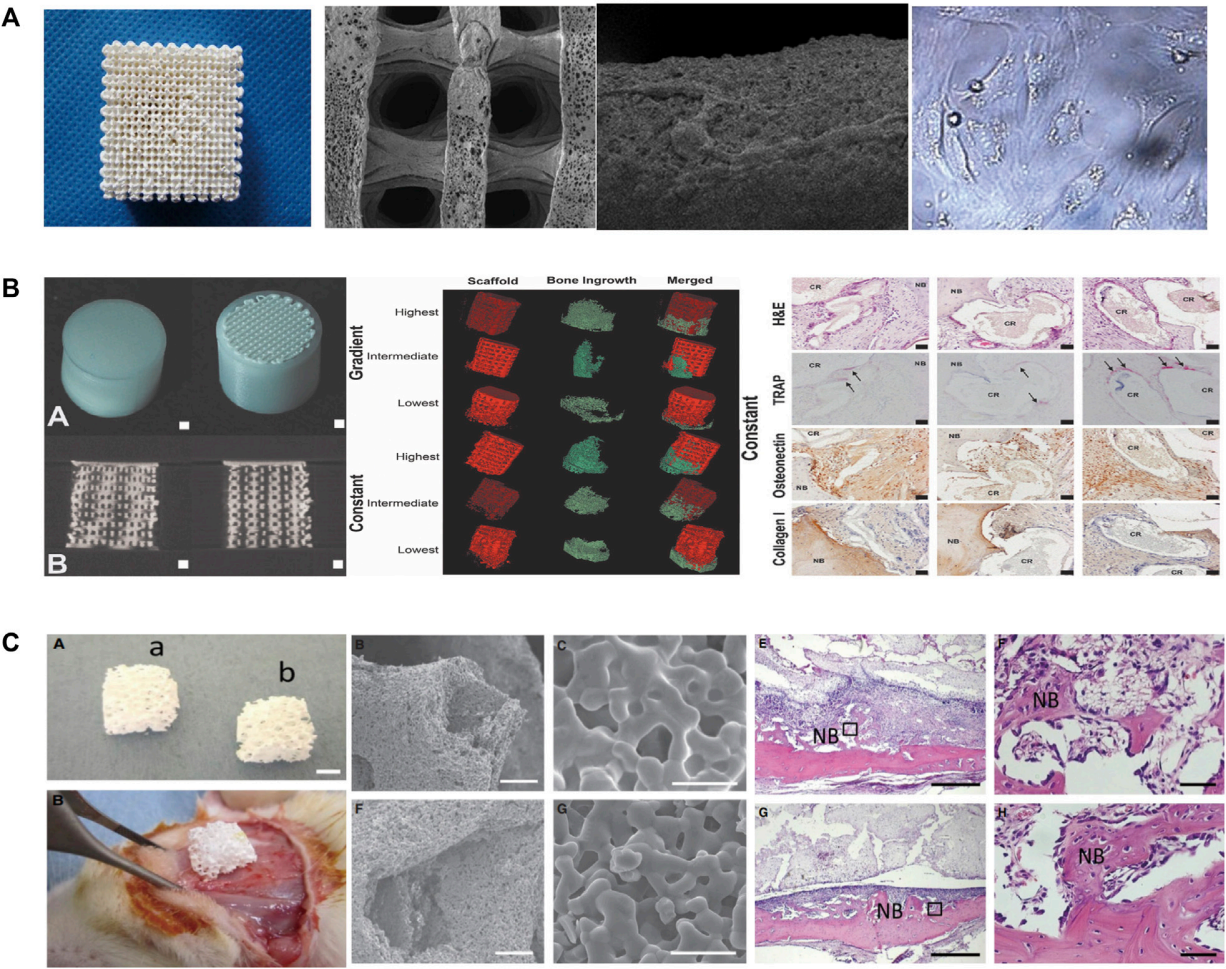
**(A)** Representative of the 3D porous nHA/PLGA composite scaffolds. SEM images of the porous PLGA/n-HA scaffolds and microscopic images of cells. **(B)** Visualization of the implant and formation of new bones via µCT. H&E, TRAP, osteonectin, and collagen type I staining of decalcified sections of porous structures. **(C)** Morphological characteristics and in vivo implantation of the scaffolds. The representative histological analysis of new bone formation after implantation. SEM, Scanning electron microscope; LDM, low-temperature deposition manufacturing; H&E, Hematoxylin and eosin; TRAP, Tartrate-resistant acid phosphatase.

The fabrication of PLLA/TCP composite scaffolds for bone tissue engineering was first presented by Xiong et al. (2002) as a new deposition manufacturing method. They mixed PLLA solution and TCP powder and finished the printing process in a low temperature environment of 0°C. The sublimation of dioxane in the freeze-drying procedure created micropores around the printed macropores. Yang et al. (2006) fabricated PLGA/TCP composited scaffolds by LDM and performed cell experiments to observe the mechanical strength, cell affinity, and degradation. Similarly, other PLGA/TCP scaffolds have been fabricated adopting double-nozzle technology and optimizing fabrication parameters to obtain the best mechanical properties and biocompatibility (Kai et al., 2009; Li et al., 2011). Lian et al. (2021) used LDM technology to develop sponge-like PLCL/HA scaffolds that promoted the interactions between MSCs and materials at a temperature of −28°C. In addition, the paracrine function of MSCs on scaffolds was improved, enhancing immunomodulation, angiogenesis, and osteogenic potential. The LDM-printed sponges with hierarchical interconnected pores could promote cell-scaffold interaction and upregulates osteogenic and vasogenic activity via the signaling paths (Figure 4A) (Lian et al., 2021). However, an *in vitro* cell experiments showed that cell viability on the surface of the scaffolds was time dependent and unaffected by the composited PLGA/nHA (Chen et al., 2013a).

#### 3.3.2 Polymer-ceramic scaffolds with hydrogels

Hydrogels and collagen have been used to increase the hydrophilicity of scaffolds composited with ceramics and polymeric materials. For example, PLGA/β-TCP scaffolds prepared by LDM have been wrapped with collagen to improve the hydrophilicity and osteogenic differentiation (Wang et al., 2012; Zhang et al., 2017; Diloksumpan et al., 2020 composited hydrogel and calcium phosphate to prepare the scaffolds at ambient temperature (20°C–25°C) and encased the scaffolds in a PCL shell (Diloksumpan et al., 2020). They found that scaffolds with constant pores had better osteogenic properties than gradient scaffolds and that bone growth was enhanced in only one direction. Figure 4B presents the formation of new bones in scaffolds and the staining of hematoxylin and eosin, tartrate-resistant acidic phosphatase, osteonectin, and collagen type I in decalcified sections of porous structures (Diloksumpan et al., 2020). Similarly, Dou et al. developed a PLGA/HA framework and filled it with gelatin through LDM (Dou et al., 2021). The combination between the scaffolds and the original tissue was closer than that for single-material ones.

#### 3.3.3 Polymer ceramic scaffolds obtained drugs or bioactive factors

Another major advantage of LDM in biomaterial application is the potential of including drugs and growth factors to improve bone healing or resist infection during the creation of polymer–ceramic composites. Dong et al. (2014) proposed a method of using solvent evaporation and low-temperature drying technology to prepare implants, which were composited with poly-DL-lactide, HA, and anti-tuberculosis drugs. Salvianolic acid B (SB) is an active component extracted from danshen and it can improve osteogenesis and angiogenesis (Cui et al., 2012). Lin et al. printed the composited scaffolds composed of PLGA, β-TCP, and SB through LDM at a temperature of −28°C and evaluated the effects on spinal fusion models (Lin et al., 2019). Icariin is another bioactive factor used to producing composite scaffolds. It is compounded into degradable scaffolds fabricated with polymer ceramic composites to provide mechanical support and promote the process of bone regeneration (Lai et al., 2018; Zhang et al., 2021). Chen et al. (2012); Chen et al. (2013b) incorporated BMP-2 and phytomolecule icaritin into PLGA/TCP scaffolds and found that icariin enhanced bone calcium deposition and regeneration.

Wang et al., 2017a printed scaffolds through LDM with materials composited of Ca-P, PLLA and BMP-2 on a custom-made cryogenic substrate at the temperature of −30°C. The scaffolds had well biological activity and sustained release of Ca^2+^ and BMP-2. Another type of diphasic magnetic scaffold was fabricated by LDM with PLGA, collagen, nano-hydroxyapatite, and Fe_2_O_3_ using a low-temperature rapid prototyping instrument at −4°C (Huang et al., 2018). It had good biocompatibility and matched better to the structure of cartilage or subchondral bones. Yoshida et al. (2015) fabricated PLGA/β-TCP scaffolds combined with fibroblast growth factor-2, which are showed good bioeffect for bone augmentation. There are other works on the loading of active factors, such as platelet-rich fibrin, magnesium, cucurbitacin B, osteogenic peptide, arginine-glycine-aspartic acid (RGD) and CaO_2_ microspheres, on scaffold materials to increase osteogenesis (Wei et al., 2015; Song et al., 2018; Lai et al., 2019; Cheng et al., 2021; Lai et al., 2022; Wang et al., 2022). It is worth mentioning that the platelet-rich fibrin facilitates hemostasis and the secretion of growth factors while degrading fibrin (Song et al., 2018). Magnesium provides mechanical properties and biodegradability for polymer-ceramic scaffolds (Lai et al., 2019). Scaffolds fabricated at −30°C had 3D porous structures and enhanced the new bone formation within the tunnel after implantation (Figure 4C) (Lai et al., 2019). Wang et al. (2022) fabricated scaffolds on a receiving platform at a temperature of −10°C and showed that CaO_2_ improves the expression of transcription factors by releasing O_2_ and thus promotes osteogenesis. RGD-containing peptides have the ability for modulation of cell adhesion and differentiation (Li et al., 2017).

### 3.4 Others

There are scaffolds that are constructed without compositing a ceramic or polymer framework. Table 5 presents bone tissue engineering scaffolds based on other materials. Lode et al. (2016) dispersed high-density collagen to stabilize structures in LDM and adopted chemical crosslinking with 1-ethyl-3-(3-dimethylaminopropyl) carbodiimide. Zhang et al. (2007) prepared scaffolds comprising collagen/chitosan/BMP-7 materials and implanted them into defects of mandible. Similarly, Reed et al. (2016) developed an acellular scaffold with chitosan/alginate and controlled the structure of the scaffold adopting LDM technology to improve cell influx and distribution. Lee et al. (2018) used decellularized extracellular matrix) to induce cellular activities and SF to achieve proper mechanical strength. The scaffolds were fabricated on a low-temperature (−40°C) stage using a 300-μm nozzle at a speed of 10 mm s^−1^. 3D-printed scaffolds have been fabricated with gelatin and SF, platelets, or polyols adopting crosslinking and freeze-drying technologies (Zhu et al., 2016; Liu et al., 2021a). He et al. (2021) combined natural modified protein technologies with LDM to fabricate scaffolds of BMP-2 and Human Bata Defensin-3 (hBD3). The scaffolds could realize bone induction through BMP-2 and antibacterial properties through hBD3. Karamat-Ullah et al. (2021) developed the silica-SF bio-ink to print scaffolds by LDM. The antibacterial peptide was covalently linked to SF and had effective bactericidal ability.

**TABLE 5:** LDM of other scaffolds for bone tissue engineering.

Year	Team	Materials	Properties of scaffolds
2007	Zhang et al	Chitosan/Collagen/BMP-7	The scaffolds had higher ALP activity and promoted the expression of osteopontin and bone sialoprotein
2016	Lode et al	Collagen	The high viscous and density collagen was used and the scaffolds were crosslinking with carbodiimide EDC.
2016	Zhu et al	Gelatin/platelets	The scaffolds had special internal porous structures for bone tissue and large molecules to infiltrate in better
2018	Lee et al	Collagen/dECM/SF	The dECM was used to induce cellular activities and SF could enhanced the mechanical strength for scaffolds
2021	He et al	PHA/BMP-2/hBD3	The scaffolds realized directional bone induction, angiogenesis and antibacterial effects at the same time
2021	Ullah et al	Silica/SF/antimicrobial peptides	The antimicrobial peptides were covalently linked to silica-SF scaffolds to show great bactericidal effects
2021	Zafeiris et al	SF/Gelatin/polyols	The scaffolds had good rheological properties and enhanced osteogenic by Samd1/5/8 and Runx2 pathways

BMP-7, bone morphogenetic protein-7; dECM, decellularized extracellular matrix; SF, silk-fibroin; PHA, phytohaemagglutinin; hBD3, human Beta Defensin 3.

## 4 Conclusion and challenges

In this article, we introduced the technical process of LDM and reviewed the applications of this technology to bone tissue engineering scaffolds. As a green manufacturing method, LDM not only controls the pore size of the scaffolds through the use of CAD tools, but also preserves the activity of biological factors. However, there remain many challenges in the LDM of scaffolds. First, the mechanical strength of a scaffold fabricated by LDM is commonly low. Although composited materials improve the mechanical properties, the strength requirements of load-bearing bone are not met. In addition, it is difficult to select a solvent that dissolves both synthetic and natural polymers. Few solvents have been used up to the point and it is undeniable that the benefits of this technology are underutilized. Secondly, it is difficult to balance the degradation of scaffold materials with the regeneration of new bone tissue. There is no doubt that such osteogenesis is not ideal when the degradation rate of scaffolds is faster than the regeneration rate of new bone. Furthermore, the majority of the degradation products are acidic and thus mildly toxic or even toxic to the human body. In addition, a suitable substrate temperature and requirements of the printing platform are not clear. In brief, materials printed on cold plates and freezers form ice crystals in different orientations, which control the microstructures of porous scaffolds. Developing a more suitable low temperature printing system is one of the future research directions.

## 5 Prospects

Bone regeneration is not just a simple process of bone formation and resorption. It is a multi-system including the musculoskeletal system and immune system (Zheng et al., 2021). In addition, the combination of antimicrobial drugs and biomaterials can effectively control bone infection and improve prognosis while promoting bone repair (Hu et al., 2021). In contrast to traditional manufacturing techniques, 3D printing, particularly LDM, has the advantage in that it can produce tissue engineering scaffolds with customized forms, bioactivity, porosity, and mechanical properties. One approach of bioprinting involves injecting cell-rich hydrogels into scaffolds, which has drawbacks including an uneven cell distribution and an injection pressure that affects cell activity and scaffold porosity. An exceptional benefit of LDM is the ability to fabricate scaffolds with linked macro-pores and micro-pores while consistently allowing the integration of biomolecules, such as live cells, into the scaffolds. In addition, LDM allows the customization of scaffolds through changing the nozzles and combining various RPM techniques.

An ideal bone tissue engineering scaffold fabricated by LDM has the following properties. The scaffolds must, first and foremost, be of high mechanical quality to suit the demands of bone tissue, particularly the stability required by load-bearing bone. Second, the material should have strong cytocompatibility and a capacity for bone conduction, vascularization, nervousness, and disintegration, among other good biological qualities. Third, the interfacial repair of complicated bone defects depends on the creation of multilayer gradient scaffolds made of bone, cartilage, and soft tissue. LDM is a promising RPM technique that promises the fabrication of ideal scaffolds and is expected to become indispensable in bone tissue engineering. The next step is to investigate various solvent systems for blending different materials or bioactive components and to develop an ideal printing workstation. Bioinks with optimal properties have been prepared by adjusting parameters to obtain a balance of mechanical properties and biological activity in scaffolds. In future work, composite scaffolds with near-ideal levels should be further prepared with LDM technology to promote the regeneration of bone tissue and surrounding tissues and ultimately realize multi-organ bioprinting.
